# Application of the multiphase optimization strategy to a pilot study: an empirical example targeting obesity among children of low-income mothers

**DOI:** 10.1186/s12889-016-3850-y

**Published:** 2016-11-22

**Authors:** Kari C. Kugler, Katherine N. Balantekin, Leann L. Birch, Jennifer S. Savage

**Affiliations:** 1 The Methodology Center, The Pennsylvania State University, 404 Health & Human Development Building, University Park, PA 16802 USA; 2 Department of Nutritional Sciences, The Pennsylvania State University, University Park, PA USA; 3 Center for Childhood Obesity Research, The Pennsylvania State University, University Park, PA USA; 4 Department of Foods and Nutrition, The University of Georgia, Athens, GA USA

**Keywords:** Factorial study design, MOST, Obesity, Children, Remotely delivered, Low income

## Abstract

**Background:**

Emerging approaches to building more efficient and effective behavioral interventions are becoming more widely available. The current paper provides an empirical example of the use of the engineering-inspired multiphase optimization strategy (MOST) to build a remotely delivered responsive parenting intervention to prevent obesity among children of low-income mothers with and without depressive symptoms.

**Methods:**

Participants were 107 mothers with (*n* = 45) and without (*n* = 62) depressive symptoms who had a child aged 12 to 42 months participating in the Women, Infants and Children program. Participants were randomized to one of sixteen experimental conditions using a factorial design that included a combination of the following eight remotely delivered intervention components: *responsive feeding curriculum* (given to all participants), *parenting curriculum*, *portion size guidance*, *obesogenic risk assessment*, *personalized feedback on mealtime routines*, *feeding curriculum counseling*, *goal setting*, *mobile messaging*, and *social support*. This design enabled efficient identification of components with low feasibility and acceptability.

**Results:**

Completion rates were high (85%) and did not statistically differ by depressive symptoms. However, mothers with depressive symptoms who received *obesogenic risk assessment* and *personalized feedback on mealtime routines* components had lower completion rates than mothers without depressive symptoms. All intervention components were feasible to implement except the *social support* component. Regardless of experimental condition, most participants reported that the program increased their awareness of what, when, and how to feed their children.

**Conclusions:**

MOST provided an efficient way to assess the feasibility of components prior to testing them with a fully powered experiment. This framework helped identify potentially challenging combinations of remotely delivered intervention components. Consideration of how these results can inform future studies focused on the optimization phase of MOST is discussed.

## Background

Childhood obesity is a public health concern that disproportionately impacts children from low-income families [1]. Without effective intervention, it is estimated that 42% of children will be obese by 2050 [2]. Modifiable factors such as responsive parenting practices have been identified as important targets for interventions in reducing overweight and rapid growth during infancy [3–5], but few early preventive interventions have ultimately had positive effects [6–10]. The few interventions that have effectively impacted child weight are generally not feasible for widespread implementation as they are intensive (i.e., require numerous in-person visits over an extended period of time) and expensive to implement because they are predominately delivered in the home. These implementation features limit feasibility with at-risk populations, including lower income households. Furthermore, the existing effective interventions have been evaluated as a package with numerous intervention components [8, 10, 11]; it remains unclear which components are most critical to preventing obesity, particularly among low-income populations.

Low-income populations experience unique challenges related to chronic stress and depression, which can negatively impact feeding and increase obesity risk among young children [12–14]. For example, mothers with depressive symptoms are more likely to use controlling feeding practices [14], exhibit reactive, hostile, or withdrawn behaviors [13], and be less sensitively attuned to their infant’s needs [15]. As a result, they may engage in or need different intervention content (e.g., [16]). The current paper describes the application of the multiphase optimization strategy (MOST), an engineering-inspired framework for the optimization of behavioral and biobehavioral interventions [17, 18], to a pilot study to identify the most feasible and acceptable components for low-income mothers with and without depressive symptoms.

MOST is a framework that includes three phases—preparation, optimization, and evaluation—and emphasizes optimization *before* evaluation of a multi-component intervention with a 2-arm randomized controlled trial (RCT) [18]. The *preparation* phase includes assessment of the literature, development of a conceptual model, identification of intervention components, and completion of pilot studies to examine the feasibility and acceptability of intervention components. At the end of this phase a clearly stated optimization criterion is identified that explicitly states the overall goal for the intervention, taking into consideration any resource constraints (e.g., participant burden, staff time, costs). *Optimization* is a process that uses fully powered, efficient, randomized experimentation to gather information about the individual and combined performance of intervention components to identify one of the best possible combinations that is effective at addressing the public health problem at hand, *subject to given constraints* (e.g., no “inactive” components, cost under $200 per participant). Finally, *evaluation* uses randomized experimentation to determine whether the optimized intervention performs better than control or standard of care.

Although MOST has not yet been used to optimize a childhood obesity intervention, in other domains (e.g., smoking cessation, weight loss maintenance) researchers have used fully powered, randomized factorial experiments to examine the performance of intervention components during the optimization phase (for examples see [19, 20]). Factorial designs are used for optimization because they provide estimates of the components’ performance, individually and in combination, which are then used for making decisions about which components should be retained in the optimized intervention (see [18] for a more detailed review). Factorial experiments consist of two or more factors, each with 2 or more levels, whose experimental conditions take on all possible combinations of the levels across all such factors (e.g., a full factorial design with 3 factors, each with two levels, yields 8 experimental conditions). An analysis of variance of a factorial design provides researchers with the information to determine which components are “active” and which are unnecessary, and which components are synergistic or antagonistic with one another. When the goal is to build an intervention that is effective, cost-effective, or scalable, it is important to isolate these effects.

To date, RCTs testing early interventions to prevent obesity have been delivered in the home, an approach that is labor intensive and expensive [21]. One way to develop a more cost-effective and scalable intervention is to modify the mode of delivery to use technology to deliver the intervention remotely. Remotely delivered interventions have been used as successfully as those delivered in person to change behaviors, but they have better reach, require less staff effort, and produce less participant burden; thus they are cost effective [21]. Although few remotely delivered interventions have been implemented with low-income populations, mobile devices are widely used by low income individuals across varying sociodemographic groups [22], indicating that these interventions might be feasible with this population.

The objective of the current study was to use MOST to assess the feasibility and acceptability of responsive parenting intervention components among low-income mothers with and without depressive symptoms and their 12 to 42 month-old children. Based on trials that have effectively reduced the incidence of childhood obesity [8], eight remotely delivered intervention components were assessed using a sample of women enrolled in the Women, Infants, and Children (WIC) program. This approach, which is still fairly novel in the behavioral sciences [17, 18], enabled us to examine the acceptability of intervention components and the feasibility of conducting a factorial experiment before investing resources into components that are not worthwhile to implement.

## Methods

### Participants

**Fig. 1:**
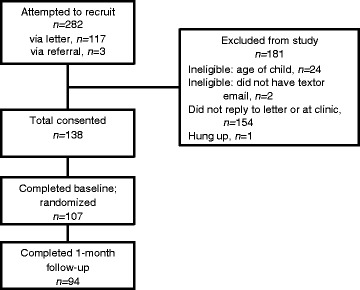
CONSORT diagram

Participants for the current study included 107 mothers (see Table 1 for sample characteristics) recruited from WIC clinics between June 2013 and April 2014. Mothers were eligible to participate in the study if they were at least 18 years of age, had a child between 12 and 42 months of age, spoke English, and had a reliable mobile phone or internet access. Prior to completion of a baseline survey, mothers provided consent (either in-person or over the phone) and were reminded that their participation in this research was confidential and that no personal identifiable information will be stored with the data (see Fig. 1 for diagram of randomization process). Upon completion of the survey, participants were randomized to an experimental condition (see Table 2). We blocked on maternal depressive symptoms (measured at baseline; Center for Epidemiological Studies Depression Scale [23]) at a threshold associated with clinical relevancy (score 16 or greater) to assess feasibility of the intervention components separately among participants with and without depressive symptoms.

**Table 1:** Maternal socio-demographic characteristics of the sample (*n* = 107)

Maternal Characteristics	%
Mean age (SD)	29.2 (6.3)
Race/Ethnicity	
White	85%
Black	8%
Hispanic	5%
Other	2%
Marital status	
Married	57%
Single	28%
Divorced/separated	8%
Not married, living with partner	8%
Employment	
Full-time	27%
Part-time	30%
Unemployed	29%
Other	15%
CESD^a^, Mean (SD)	13.2 (12.2)

^a^ *CESD* Center for Epidemiologic Studies on Depression (0–60) [23]

**Table 2:** Experimental conditions (On = received intervention materials; *Off* = did not receive intervention materials)

Experimental condition	Responsive feeding	Parenting curriculum	Portion size	Obesogenic risk assessment	Mealtime routines	Social support	Responsive feeding counseling	Mobile messaging	Goal setting
1	On	*Off*	*Off*	*Off*	*Off*	*Off*	*Off*	*Off*	*Off*
2	On	*Off*	*Off*	*Off*	On	On	On	On	*Off*
3	On	*Off*	*Off*	On	*Off*	On	On	*Off*	On
4	On	*Off*	*Off*	On	On	*Off*	*Off*	On	On
5	On	*Off*	On	*Off*	*Off*	On	*Off*	On	On
6	On	*Off*	On	*Off*	On	*Off*	On	*Off*	On
7	On	*Off*	On	On	*Off*	*Off*	On	On	*Off*
8	On	*Off*	On	On	On	On	*Off*	*Off*	*Off*
9	On	On	*Off*	*Off*	*Off*	*Off*	On	On	On
10	On	On	*Off*	*Off*	On	On	*Off*	*Off*	On
11	On	On	*Off*	On	*Off*	On	*Off*	On	*Off*
12	On	On	*Off*	On	On	*Off*	On	*Off*	*Off*
13	On	On	On	*Off*	*Off*	On	On	*Off*	*Off*
14	On	On	On	*Off*	On	*Off*	*Off*	On	*Off*
15	On	On	On	On	*Off*	*Off*	*Off*	*Off*	On
16	On	On	On	On	On	On	On	On	On

### Study design

A fractional factorial design was used. The benefit of this design is that the number of experimental conditions is reduced in a principled way, lessening the burden of implementing more experimental conditions, compared to a full factorial (see [17] and [24] for greater detail about fractional factorial experiments). A full factorial design with eight different intervention components, each with two levels (i.e., *On* = receive the component and *Off* = do not receive the component), would require 2^8=^256 experimental conditions. However, with our fractional factorial design, only 1/16 were implemented (256/16 = 16 experimental conditions; see Table 2). We chose this fractional factorial design with sixteen experimental conditions (as opposed to 32 or 64) because this was a manageable number of experimental conditions to execute for a single research staff member; further, dividing our sample by sixteen resulted in six to seven participants per condition, or three to four with and without depressive symptoms. Having six to seven participants per condition allowed us to qualitatively assess aspects of feasibility, implementation quality, and program satisfaction; the concern about aliasing of effects (disentangling of effects that are bundled together because of the experimental conditions that are removed) in a fractional factorial design was not an issue given our focus on feasibility rather than effectiveness.

A factorial design program (SAS PROC FACTEX) was used to determine which experimental conditions were to be used to give the strongest design and ensuring balance across the conditions. For each intervention component (except the responsive feeding curriculum component, which was given to everyone), eight experimental conditions are *On* and eight experimental conditions are *Off*. For example, for the parenting curriculum component, there are eight experimental conditions that are *On* (i.e, conditions #9-16) and eight conditions that are *Off* (i.e, conditions 1-8). Within the experimental conditions where the parenting curriculum is *On*, every other intervention component has four conditions that are *On* and four conditions that are *Off*. This balancing feature is one of the unique characteristics of a factorial design that lends itself to efficiency and generalizability [25].

### Intervention components

The intervention components were drawn from other intervention studies such as SLIMTIME [10] and INSIGHT [7] and clinical experience.

### Responsive feeding curriculum

All experimental conditions were mailed the responsive feeding curriculum handout, which was informed by prior research [7, 10] and included age-appropriate guidance on establishing routines and limits around feeding, the division of feeding responsibility [26], alternatives to using food as a reward, snacking, juice consumption, and picky eating.

### Parenting curriculum

Participants were mailed the parenting guidance handouts on active social play (e.g., “Do not have the TV on during meals with your toddler”), sleep (e.g., “Start a bedtime routine 20–45 min before you want to put your toddler to bed”), and emotional self-regulation (e.g., tips for preventing and stopping tantrums.).

### Portion size guidance

Participants were mailed a toddler-appropriate portion plate [27] with space for appropriate portions of fruits, vegetables, meat, and grains; a set of measuring cups; and a handout on age-appropriate portions, including serving size recommendations, portion size tips (e.g., 1 tablespoon = the size of your thumb), and tips for increasing intake of fruits and vegetables [28].

### Obesogenic risk assessment

Participants were given personalized, yet scripted feedback, based on their responses to the Family Nutrition and Physical Activity questionnaire [29], which was modified to be age appropriate for toddlers. Research staff provided feedback over the phone during week 2.

### Personalized mealtime routine feedback

Participants completed a two-day food record for their child on which they recorded the time of the day food was served, whether it was a snack or meal, type of food or drink consumed, and location. Participants were given tailored feedback (week 4) about ways to improve mealtime routines and set limits for feeding a toddler.

### Responsive feeding counseling

Research staff called participants to review and discuss the responsive feeding curriculum over the phone. This was also an opportunity for research staff to answer feeding-related questions while highlighting the key points (e.g., “It is your job as a parent to decide *when* and *what* to serve your child, and then your child should be able to decide *what* and *how much* to eat.”). Participants reviewed this material during a phone call during week 2.

### Social support

Participants were asked to identify a support person (i.e., anybody to whom they would go to get advice about their child(ren)) and give them a consent form to allow research staff to contact them. Once the support person returned the consent form, they were mailed the responsive feeding handout. Two to three days later (typically during week 2), research staff called the support person to go over the responsive feeding curriculum, highlighting the same key points conveyed to the mother.

### Mobile messaging

Text messages (6 nutrition texts and 2 child development texts) and video messages related to feeding a toddler (e.g., picky eating, snacking) were sent via phone or email. An example text message was, “You are a great role model! Snack on fruits and vegetables together at meal time.” The mobile messaging components were designed as boosters to the responsive feeding curriculum. Messages were sent three times per week for the duration of the intervention.

### Goal setting

Participants were mailed a list of 13 nutrition-related goals (e.g. “If your toddler is thirsty between meals, offer water. Limit juice to 4 oz or less a day and serve 100% fruit juice.”). During each phone call (weeks 2 and 4), participants chose a goal to work on over the next 2 weeks. Research staff assisted the participant in selecting a goal and outlining behavior change strategies necessary to achieve that goal. During the second phone call (week 4), research staff discussed progress towards the first goal, providing trouble-shooting tips if the goal hadn’t been met. A second goal was then selected to work on over the next two weeks.

### Intervention implementation

The intervention experience varied across participants based on their randomization to one of the sixteen different experimental conditions; participants received different combinations of intervention components, which varied in dose, number of contacts, and duration. All components were remotely delivered (e.g., via mail, email, text, or phone) by primarily one research staff member trained in health education. The timing, mode of intervention delivery, and data collection time points are shown in Table 3. To ensure fidelity of each intervention component, a computer-generated intervention scheduler was created for each participant to alert research staff to conduct phone calls, mail intervention materials, send reminder text messages, send mobile messages, and administer surveys and conduct a semi-structured phone interview immediately following the intervention. Research staff used a script for each phone-delivered component.

**Table 3:** Study design overview

	Week 0	Week 1	Week 2	Week 3	Week 4	Week 5
Data collection Intervention components	Online/paper					Online/paper
Responsive feeding curriculum		Mailed				
Parenting curriculum		Mailed				
Portion size guidance		Mailed				
Obesogenic risk assessment			Phone			
Personalized feedback on mealtime routines					Phone	
Social support			Phone			
Responsive feeding counseling			Phone			
Mobile messaging		Text/email	Text/email	Text/email	Text/email	
Goal setting			Phone		Phone	

### Outcomes

The primary outcomes of the study were feasibility and acceptability of the intervention components and feasibility of implementing a factorial study design as part of a pilot study. Traditional approaches to examining feasibility and acceptability of an intervention are still relevant for a factorial design (e.g., tracking completion rates, attrition, intervention implementation and fidelity, and staff and participant acceptability). However, these approaches can be expanded to allow researchers to see obvious differences between specific combinations of intervention components, even when power in the pilot study is not sufficient to reach statistical significance.

To measure feasibility, overall completion rates were tracked for each intervention component. In addition, the number of times that research staff attempted to contact a participant via phone or text message was recorded. After each phone call, staff reported the duration of the call and the degree to which the participant was engaged, rated on a scale from (1) *rarely engaged* (participant did not sustain active engagement during this session) to (4) *highly engaged* (participant demonstrated a high level of active engagement throughout the session). To measure program satisfaction, a semi-structured phone interview was conducted immediately following the intervention. Participants answered questions about the components they received, in addition to open-ended questions about implementation and preferred mode of delivery. No formal qualitative methods (e.g., content analysis) were used to examine the responses to the open-ended questions. Since one of the aims of the study was to test the feasibility and acceptability of intervention for mothers with and without depressive symptoms, all the results explored whether there were statistically significant differences based on depressive symptoms, even though the study was not specifically powered to detect these differences (at power = .80, able to detect *d* ≥ .63, with 40 participants/group). The post-intervention phone calls were transcribed, double-entered, and coded. All study procedures were approved by the university’s Institutional Review Board.

## Results

### Feasibility

Overall, completion rates were high: among those assigned a phone intervention component, 88% of participants completed their first call, and 81% of participants assigned to a second call completed it. There were no statistically significant differences in completion rates based on depressive symptoms (1^st^ call, *p* = .29; 2^nd^ call, *p* = .13). For the first call, it took on average 2 back/forth (i.e., research staff called and left a message, participant called back) contacts (range: 1 to 6) to reach the participant; there were no differences by depressive symptoms (*p* = .52). The average duration of the first call was 13 min and ranged from 3 to 50 min, which reflects both the variability in the number of components (1 to 4) that participants were assigned to and the loquacity of the participant; call duration did not differ by depressive symptoms (*p* = .47). The second call also required an average of 2 back/forth contacts (range: 1 to 6). This approached significance by depressive symptoms (*p* = .09), with slightly more contacts required for participants with depressive symptoms (2.1) than for participants without (1.8). The second call was an average of 10 min long (range 2 to 25 min); call duration did not differ by depressive symptoms (*p* = .84).

The use of a factorial experiment enabled us to assess whether women would differentially participate in different combinations of intervention components or whether any particular combination created extra burden. Although this study was not powered to examine the effect of these differences, as shown in Table 4, we observed that second call completion rates were lowest for participants with depressive symptoms who were randomized to experimental condition 4 (received responsive feeding curriculum, obesogenic risk assessment, personalized feedback on mealtime routines, mobile messaging, and goal setting components) and experimental condition 12 (received responsive feeding curriculum, parenting curriculum, obesogenic risk assessment, personalized feedback on mealtime routines, and responsive feeding counseling components), with average rates of 33% and 50%, respectively. Two components—obesogenic risk assessment and personalized feedback on mealtime routines—were common across these experimental conditions. Most of the other experimental conditions with these components also had lower completion rates (50–67%), particularly among participants with depressive symptoms.

**Table 4:** Participation rates across experimental condition (stratified by baseline depressed status)

				1^st^ call (*N = 83/94*)		2^nd^ call *(N = 64/79*)		
Experimental condition	Overall sample (*N*)	No symptoms^a^ mean CESD (*N* = 62)	Symptoms mean CESD (*N* = 45)	No symptoms completed	Symptoms completed	No symptoms completed	Symptoms completed	Overall completion
1	7	7.0	29.0	n/a^b^	n/a	n/a	n/a	n/a
2	7	6.0	29.7	4/4 (100%)	3/3 (100%)	4/4 (100%)	2/3 (67%)	13/14 (93%)
3	7	3.0	31.0	5/5 (100%)	2/2 (100%)	4/5 (80%)	2/2 (100%)	13/14 (93%)
4	6	2.5	26.0	1/3 (33%)	2/3 (67%)	3/3 (100%)	1/3 (33%)	7/12 (58%)
5	6	8.2	16.0	5/5 (100%)	1/1 (100%)	5/5 (100%)	1/1 (100%)	12/12 (100%)
6	6	7.7	36.0	2/3 (67%)	3/3 (100%)	2/3 (67%)	3/3 (100%)	10/12 (83%)
7	7	3.7	23.3	1/3 (33%)	4/4 (100%)	n/a	n/a	5/7 (71%)
8	6	5.3	19.5	3/3 (100%)	3/3 (100%)	3/3 (100%)	2/3 (67%)	11/12 (92%)
9	7	8.7	23.7	4/4 (100%)	3/3 (100%)	3/4 (75%)	3/3 (100%)	13/14 (93%)
10	7	8.3	27.8	3/3 (100%)	4/4 (100%)	3/3 (100%)	4/4 (100%)	14/14 (100%)
11	7	7.8	21.5	4/5 (80%)	2/2 (100%)	n/a	n/a	6/7 (86%)
12	7	8.7	29.3	2/3 (67%)	3/4 (75%)	2/3 (67%)	2/4 (50%)	9/14 (64%)
13	7	7.2	27.0	4/5 (80%)	2/2 (100%)	n/a	n/a	6/7 (86%)
14	6	5.0	38.5	n/a	n/a	3/4 (75%)	1/2 (50%)	4/6 (67%)
15	7	6.8	22.0	5/5 (100%)	1/2 (50%)	4/5 (80%)	1/2 (50%)	11/14 (78%)
16	7	9.5	29.3	4/4 (100%)	3/3 (100%)	4/4 (100%)	2/3 (67%)	13/14 (93%)
Total	*N* = 107	6.7 (3.6)	28.4 (11.2)	47/55 (85%)	36/39 (92%)	40/46 (87%)	24/33 (73%)	147/173 (85%)

^a^No depressive symptoms was calculated as CESD score <16 = No depressive symptoms; CESD ≥ 16 = Depressive symptoms (Center for Epidemiologic Studies on Depression) [23]

^b^n/a = Not applicable due to randomization scheme

### Participant engagement

Most of the participants were rated by research staff to be moderately to highly engaged during their phone calls (1^st^ call, mean = 3.9; 2^nd^ call, mean = 3.8, on a scale of 1 to 4). There were no statistically significant differences in mean engagement by depressive symptoms for the first call (mean engagement = 3.9 for each group; *p* = .71). However, the difference approached significance for the second call (*p* = .06); participants with depressive symptoms were slightly less engaged than those without depressive symptoms (mean 3.6 vs 3.9, respectively). Specifically, participants with depressive symptoms were less engaged than participants without depressive symptoms in experimental condition six (received responsive feeding curriculum, portion size, personalized feedback on mealtime routines, responsive feeding counseling, and goal setting components) and experimental condition eight (responsive feeding curriculum, portion size, obesogenic risk assessment, personalized feedback on mealtime routines, and social support components); mean engagement was 3.0 for each. Two components common to these experimental conditions were portion size and personalized feedback on mealtime routines.

### Component acceptability

Seventy percent of participants reported spending 10 or more minutes reading through the responsive feeding curriculum. Half of the participants who received the parenting curriculum spent 10 or more minutes reading that handout, with less than 10% spending 5 min or less. All the participants randomized to receive portion size guidance reported using it at least one time per week, with 85% reporting that they used it two or more times per week. Two-thirds of women said they changed the amount of food they gave their toddler after using the portion plates and cups. Among participants who were randomized to receive obesogenic risk assessment, 71% reported making changes in their home based on the recommendations and suggestions. Among the participants who were randomized to the personalized feedback on mealtime routines component, 90% of the participants found the mealtime routine information about the timing of meals and snacks to be helpful. Among participants who received mobile messaging, a component intended as a booster to the responsive feeding curriculum, two-thirds of participants said they learned something new the text and/or video messages. Finally, among the participants who were randomized to receive the *goal setting* component, 76% liked setting goals for their family.

### Program satisfaction

During the phone interview immediately following the intervention, participants responded to a series of open-ended questions about what they liked and disliked about the program. When asked about the best part of the program, many participants reported that it increased their awareness of what they were feeding their children. For example, “I was more aware of what I was feeding her.” Other participants liked learning about routines: “I would have to say getting the routine down. Like changing him over from the juice to the water in between meals.” The few participants who identified things that they liked least about the program generally commented on data collection (e.g., “The surveys were too long”). When asked what they felt would be the most helpful to other WIC mothers, many mothers commented on the guidance of alternatives to feeding to soothe during tantrums. For example, one mother said, “Maybe the whole tantrum thing with…. [giving food to stop a tantrum], it’s just unnecessary, it causes obesity, like a lot of parents just give their kids whatever they want to shut them up.”

### Intervention implementation

Feedback from research staff indicated that the use of a randomization scheduler was critical to implementing the sixteen different experimental conditions (which varied in dose and timing) as intended. All participants who were randomized to receive mailed intervention materials reported receiving them. However, difficulties in implementation were noted for certain intervention components. Twenty percent of the participants receiving mobile messages reported technical difficulties opening the videos. The personalized feedback on mealtime routines component required participants to fill out a two-day food record and mail it back in a pre-paid envelope. Although 75% of the participants randomized to this component completed the form, fewer participants with depressive symptoms were likely to complete the form than participants without (68% vs. 81%, respectively; *p* = .26). Despite the majority of the participants identifying a support person, the support person component was difficult to implement. Less than half (41%) of the participants’ support person returned the consent form, and of these support people, less than half (45%) reviewed the responsive feeding curriculum on a phone call with research staff. Participants with depressive symptoms were less likely to have a support person complete the consent form than participants without (29% vs. 48%, respectively; *p* = .14). Almost all of the participants reported liking the remote delivery of intervention components. When asked about preference for delivery mode, one participant indicated they would have preferred in-person delivery; nearly half of all participants preferred to get materials sent to them in the mail, and the other half either preferred email or phone.

## Discussion

These findings illustrate how the principles of MOST can be used to inform the design of a responsive parenting intervention to prevent early childhood obesity among low-income families. The use of a fractional factorial design provided evidence regarding the feasibility and acceptability of potential intervention components. Without this design, we would not have been able to examine potentially challenging combinations of components that may have reduced engagement in the intervention, particularly among participants with depressive symptoms. We were able to identify components that should be refined or considered for exclusion in a future study, especially when in combination with other components. These pilot data are particularly useful when building an intervention that is focused on being effective without excessive participant burden. Further, the data generated from this pilot study (as part of the preparation phase of MOST), provide preliminary findings that can be used to inform a later fully powered study to build an optimized intervention.

Among groups receiving different combinations of the eight intervention components, the completion rates were lowest for participants who received both obesogenic risk assessment and personalized feedback on mealtime routines components, and these rates were even lower for participants with depressive symptoms. The personalized feedback on mealtime routines component required participants to fill out paperwork right after each meal so that they could receive customized obesogenic prevention messaging. Methods used to collect data to personalize these messages may have overwhelmed participants, particularly those with depressive symptoms, decreasing retention [30, 31]. It is unknown whether the use of mobile applications would decrease this burden; future studies could explore this mode of delivery. Further, participants with depressive symptoms who were randomized to experimental conditions that contained both portion size guidance and personalized feedback on mealtime routines were slightly less engaged during their phone call. A feature common to these components is the element of having a routine (e.g., to use a portion plate), and participants with depressive symptoms might not have been as receptive to those messages or to using something new; however, the acceptability of the portion plate was high and suggests this might be a worthwhile component to retain as long as the personalized feedback on mealtime routines component is not included. In short, the use of a factorial design generates hypotheses about possible challenging combinations between components that can later be tested in fully powered studies to better understand the exact mechanism of these combinations.

This empirical example demonstrates that it was possible to implement a factorial design given available resources within the context of a pilot study. As noted by [17], the use of MOST does not require more resources than the classical approach, just a realignment of resources. Applied to the empirical study, this required that staff training in implementing sixteen different experimental conditions with fidelity; staff reported that a randomization scheduler and scripts were critical to ensuring the experimental conditions were implemented as intended.

In terms of building a cohesive body of literature to inform the development of effective childhood obesity prevention program among low-income mothers, the findings identified some promising intervention components for inclusion in future studies. Of the eight remotely delivered intervention components under consideration, a handful appear to be viable options for low-income mothers with and without depressive symptoms, including, feeding counseling, mobile messaging, goal setting, guidance using age-appropriate portion plates. The acceptability of these components was high, and staff reported that they were feasible to implement. However, they need to be considered in combination with one another. As noted above, many of the participants reported that the use of a portion plate changed how much food they fed their children. This could be a cost-effective strategy ($8/plate) to help teach parents proper portion sizes for their children, but this also could be an extra burden on the participants with depressive symptoms who also receive personalized feedback on mealtime routines.

Mobile messaging with low-income populations holds promise for remotely delivered interventions to have greater reach and is another cost-effective intervention strategy. The messages were well received by the participants and did not appear to be problematic when delivered in combination with other components; however, 20% of participants who received mobile messaging reported difficulty viewing the videos. In the current study, allowing participants to receive messages via email rather than text message appeared to be a reasonable work-around solution. If the difficulty of viewing the messages was due to limitations in data packages (i.e., cannot exceed 2.0 GB of data/month), then a future study could be designed to find the most effective dose of mobile messaging that does not exceed 2.0 GB of data per month. The MOST framework is well-suited to answer these types of questions.

The decision to include a component related to social support was based on evidence that s among low-income populations, positive social support can positively impact parenting behavior [32]. However, as designed, this component was not feasible to implement; research staff only talked to 18% of the support people. Our findings suggest that if this component is retained, other approaches are needed to consent and engage social support persons. For example, a mobile application or online consent procedure could ease this burden. More research is needed to effectively recruit and engage support persons in remotely delivered interventions among low-income women.

## Conclusion

Innovative approaches such as MOST are increasingly being used to build more effective multi-component behavioral and biobehavioral interventions; however, this approach has yet to be fully realized in the context of pilot studies. While the classical approach to intervention development includes conducting pilot studies, the guiding principles of MOST, in particular the resource management principle, suggests the use of alternative study designs (other than the 2-arm RCT) to acquire more information about the feasibility and acceptability of intervention components individually and in combination. Thus the results from this pilot study add to the body of preliminary data for remotely delivered childhood obesity interventions; however, additional pilot work is needed to refine the promising components to determine whether the they can be implemented over a longer time frame and to identify an optimization criterion for a future fully powered study to build an optimized childhood obesity intervention for low-income populations.
